# Ticagrelor Conditioning Effects Are Not Additive to Cardioprotection Induced by Direct NLRP3 Inflammasome Inhibition: Role of RISK, NLRP3, and Redox Cascades

**DOI:** 10.1155/2020/9219825

**Published:** 2020-08-03

**Authors:** Claudia Penna, Manuela Aragno, Alessia Sofia Cento, Saveria Femminò, Isabella Russo, Federica Dal Bello, Fausto Chiazza, Debora Collotta, Gustavo Ferreira Alves, Massimo Bertinaria, Elisa Zicola, Valentina Mercurio, Claudio Medana, Massimo Collino, Pasquale Pagliaro

**Affiliations:** ^1^Department of Clinical and Biological Sciences, University of Turin, Turin, Italy; ^2^Department of Molecular Biotechnology and Health Sciences, University of Turin, Turin, Italy; ^3^Department of Drug Science and Technology, University of Turin, Turin, Italy; ^4^Department of Translational Medical Sciences, Federico II University, Naples, Italy

## Abstract

Inhibition of either P2Y12 receptor or the nucleotide-binding oligomerization domain- (NOD-) like receptor pyrin domain containing 3 (NLRP3) inflammasome provides cardioprotective effects. Here, we investigate whether direct NLRP3 inflammasome inhibition exerts additive effects on myocardial protection induced by the P2Y12 receptor antagonist Ticagrelor. Ticagrelor (150 mg/kg) was orally administered to rats for three consecutive days. Then, isolated hearts underwent an ischemia/reperfusion (30 min ischemia/60 min reperfusion; IR) protocol. The selective NLRP3 inflammasome inhibitor INF (50 *μ*M) was infused before the IR protocol to the hearts from untreated animals or pretreated with Ticagrelor. In parallel experiments, the hearts isolated from untreated animals were perfused with Ticagrelor (3.70 *μ*M) before ischemia and subjected to IR. The hearts of animals pretreated with Ticagrelor showed a significantly reduced infarct size (IS, 49 ± 3% of area at risk, AAR) when compared to control IR group (69 ± 2% of AAR). Similarly, *ex vivo* administration of INF before the IR injury resulted in significant IS reduction (38 ± 3% of AAR). Myocardial IR induced the NLRP3 inflammasome complex formation, which was attenuated by either INF pretreatment *ex vivo*, or by repeated oral treatment with Ticagrelor. The beneficial effects induced by either treatment were associated with the protective Reperfusion Injury Salvage Kinase (RISK) pathway activation and redox defence upregulation. In contrast, no protective effects nor NLRP3/RISK modulation were recorded when Ticagrelor was administered before ischemia in isolated heart, indicating that Ticagrelor direct target is not in the myocardium. Our results confirm that Ticagrelor conditioning effects are likely mediated through platelets, but are not additives to the ones achieved by directly inhibiting NLRP3.

## 1. Introduction

Ischemic heart disease remains the leading cause of morbidity in the Western world, and the number of deaths from acute myocardial infarction (AMI) is also rapidly rising in the developing world. Although restoration of early blood flow to the ischemic myocardium with thrombolysis is presently the most effective therapy to limit infarct size, reperfusion alone is inadequate to salvage the damaged myocardium and may result in myocardial ischemia/reperfusion (IR) injury, which is characterized by excessive oxidative stress and inflammatory response [1–3]. In fact, as shown by both preclinical and clinical studies, the excess myocardial cell death resulting from the restoration of blood and oxygen supply can contribute up to 50% of the final infarct size [1–3].

In clinical practice, P2Y12 adenosine disphosphate (ADP) receptor antagonists are standard of care in AMI patients undergoing primary percutaneous intervention. Several preclinical studies have convincingly shown that these drugs significantly protect against IR injury, suggesting that these pleiotropic effects could be even more important than their antiaggregant properties in this specific clinical setting [4–8]. Clinical trials have shown that the nonthienopyridine P2Y12 antagonists such as Ticagrelor and Cangrelor were associated to lower incidence of cardiovascular mortality, AMI, or stroke compared with the thienopyridine P2Y12 antagonists, Clopidogrel and Prasugrel [9]. These differences have been ascribed, at least in part, to better and more consistent pharmacokinetic profile of the nonthienopyridine P2Y12 antagonists (Ticagrelor and Cangrelor) that do not require hepatic P450-mediated metabolic conversion of the prodrug (e.g., Clopidogrel and Prasugrel) into active forms to ensure P2Y12 receptor inhibition. Moreover, Ticagrelor is the only P2Y12 antagonist that increases tissue adenosine levels *via* inhibition of the equilibrative nucleoside transporter 1 (ENT1) by protecting the extracellular adenosine from intracellular metabolism [10–12]. This effect has been suggested to further contribute to the drug-induced cardioprotection [13–15], despite a recently published paper clouding this hypothesis [8].

Although different cell types (including endothelial cells [16]) express P2Y12 receptors, the conditioning effect of P2Y12 receptor-inhibitors has been attributed to the modulation of platelet sphingosine kinase activity and perhaps to sphingosine 1-phosphate (S1P) release [5, 17]. Since P2Y12 antagonists reduce infarct size but do not eliminate it, some other processes must be responsible of residual IR injury. Indeed, additive cardioprotective effects have been demonstrated by the combination of Ticagrelor and Rosuvastatin [13]. More recently, Audia et al. [4] demonstrated that a highly selective caspase-1 inhibitor provides additional and sustained infarct size reduction when added to Ticagrelor in preclinical models of IR injury. Caspase-1 activation is a critical choke point for eliciting activation of the inflammatory cascade NLRP3 (NOD-like receptor family, pyrin domain-containing3) inflammasome. The NLRP3 inflammasome is a large multimeric protein complex which interacts with an apoptosis-associated speck-like protein including a caspase recruitment domain (ASC), thus recruiting and activating caspase-1, which in turn mediates the cleavage of inactive prointerleukin- (IL-) 1𝛽 and IL-18 into their active forms [18]. We and others have previously demonstrated the pivotal role of the NLRP3 inflammasome in cardiometabolic disorders, including myocardial ischemia reperfusion injury, [19–23] and several NLRP3 inhibitors, including the small molecule INF we recently developed, have been tested in animal model of IR injury, showing salvage of part of the myocardium at risk [24, 25]. The cardioprotective role of NLRP3 inhibitors is attributable, at least in part, to their ability to modify protective pathways and redox environment of cells [24, 26].

In the present study, we evaluate (1) the ability of Ticagrelor and INF, alone and in combination, to reduce infarct size following IR injury, (2) the potential mechanisms of cross-talk between the two drug treatments underlying their myocardial protection, and (3) the relevance of the presence of blood in mediating cardioprotective effects and the platelet mediators released after Ticagrelor exposure.

## 2. Materials and Methods

### 2.1. Ex Vivo Rat Model of Heart IR Injury

Male Wistar rats (Harlan Laboratories, Udine, Italy) 5–6 months old, reaching a body weight of 450–550 g, were anesthetized with sodium pentothal (50 mg/kg) by intraperitoneal injections and heparinized (800 U/100 g b.w., i.m.) before being culled by cervical dislocation. The hearts were then rapidly excised, placed in an ice-cold buffer solution, and weighed. The excised hearts were rapidly perfused by the Langendorff technique with Krebs-Henseleit bicarbonate buffer containing (mM) NaCl 118, NaHCO_3_ 25, KCl 4.7, KH_2_PO_4_ 1.2, MgSO_4_ 1.2, CaCl_2_ 1.25, and Glucose 11. The buffer was gassed with 95% O_2_ : 5% CO_2_. The hearts were perfused in constant flow mode to achieve a perfusion pressure of about 80 mmHg. To assess the conditions of experimental preparation, coronary perfusion pressure was monitored during all experiments [27], and flow rate was checked in a specific time period. The temperature of the perfusion system was maintained at 37°C. After a 30 min stabilization period, the hearts were subjected to a protocol of IR, which consisted in 30 min of global no-flow, normothermic ischemia followed by a period of 60 min of reperfusion. At the end of perfusion period, the hearts were rapidly removed from the perfusion apparatus and divided in two parts by a coronal section (perpendicular to the long axis). The apical part of the left ventricle (LV, less than 1/3 of ventricular mass) was frozen rapidly in liquid nitrogen and stored at -80°C and subsequently used for Western blot analysis; the basal part of the LV was used for infarct size assessment.

The protocol was approved by the Institutional Animal Care and Use Committee of the University of Turin and conformed to the European Directive 2010/63/EU on the protection of animals used for scientific purposes.

### 2.2. Drug Treatments

**Figure 1:**
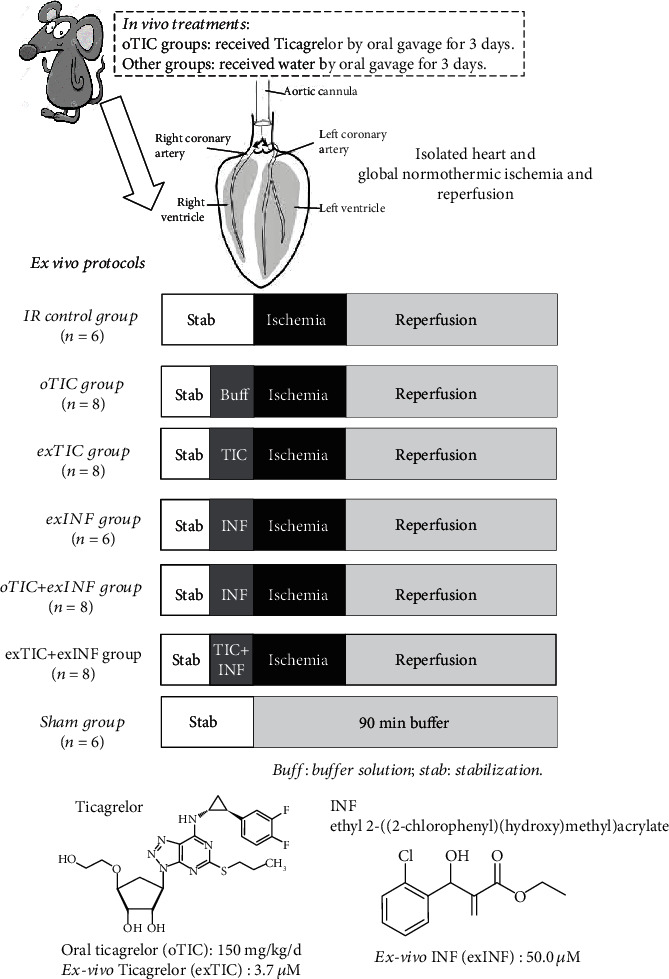
Schematic representation of rat treatments *in vivo* and various protocols *ex vivo*. Rats received water or Ticagrelor (TIC) by oral gavage for 3 days; then, hearts were isolated and perfused. After stabilization, isolated hearts were submitted to specific treatment and then to global ischemia/reperfusion protocol.

Rats (*n* = 6 − 8 per group) received water or Ticagrelor (TIC, 150 mg/kg/d) by oral gavage for 3 days (oTIC). Then, the isolated hearts were submitted to ischemia/reperfusion as described above (IR and oTIC groups). A subgroup of isolated hearts from oTIC rats were exposed to the selective NLRP3 inflammasome inhibitor INF (50 *μ*M) in the perfusate for 20 min before ischemia (oTIC+exINF). In a subsequent series of experiments, the isolated hearts from control rats were pretreated with 3.70 *μ*M Ticagrelor or 50 *μ*M exINF or both in the perfusate for 20 min before ischemia (exTIC, exINF, and exTIC+exINF groups, respectively). After stabilization, sham hearts underwent 90 min perfusion only and served as control group (Figure 1).

A stock solution of 200 mM INF in DMSO was prepared and was then diluted at a final concentration of 50 *μ*M in the perfusion buffer. The description of the synthesis of the inhibitor as well as the *in vitro* biological effects has been already published. INF is an acrylate derivative originally synthesized by Cocco et al. [28] and selected, among the tested compounds, as the most effective inhibitor of NLP3 activation (IC_50_ of 1.26 × 10^−7^ M and 1.58 × 10^−7^ M in LPS/ATP-triggered and LPS/nigericin-triggered pyroptosis, respectively). As previously documented [29, 30], INF inhibits the NLRP3 ATPase activity of isolated human-recombinant NLRP3 protein as well as caspase-1 activation, and it acts as covalent NLRP3 inhibitor through irreversible binding to nucleophilic residues present in NLRP3, with a reactivity of 0.824 ± 0.017 M^−1^ s^−1^, measured as second-order rate constant (*k*_2_) for the reaction with cysteamine. Ticagrelor was dissolved at 3.70 *μ*M concentration in Krebs solution. The *in vivo* dose of Ticagrelor and the *in vitro* concentrations of both Ticagrelor and INF were chosen according to previous studies demonstrating their efficacy against myocardial IR injury [13, 24, 28, 31].

### 2.3. Infarct Size Assessment

Infarct areas were assessed at the end of the 60 min reperfusion with the nitro-blue-tetrazolium (NBT) technique. The basal part of the left ventricle was dissected by transverse sections into two/three slices. Following 20 min of incubation at 37°C in 0.1% solution NBT (Sigma-Aldrich, St. Louis, MO, USA) in phosphate buffer, unstained necrotic tissue was carefully separated from stained viable tissue by an independent observer, who was unaware of the protocols. Since the ischemia was global and we analyzed only the basal part of the ventricle, the necrotic mass was expressed as a percentage of the analyzed ischemic tissue [32].

### 2.4. Preparation of Tissue Extracts

As previously described [33], the heart apex was homogenized at 10% (*w*/*v*) in a Potter-Elvehjem homogenizer (Wheaton, NJ, USA) using a homogenization buffer (containing 20 mM HEPES, pH 7.9, 1 mM MgCl_2_, 0.5 mM EDTA, 1 mM EGTA, 1 mM dithiothreitol (DTT), 0.5 mM phenylmethyl sulphonyl fluoride (PMSF), 0.5% Nonidet P-40, phosphatase, and protease inhibitors) and centrifuged at 1300 × g for 5 min at 4°C. To obtain the cytosolic fraction, supernatants were removed and centrifuged at 16000 × g at 4°C for 40 minutes. The pelleted nuclei were resuspended in extraction buffer containing 20 mM HEPES (pH 7.9), 1.5 mM MgCl2, 420 mM NaCl, 0.2 mM EDTA, 20% glycerol, 1 mM EGTA, phosphatase, and protease inhibitors and incubated in ice for 30 minutes followed by centrifugation at 16000 × g for 20 min at 4°C. The resulting supernatants containing nuclear proteins were carefully removed, and protein content was determined on both nuclear and cytosolic extracts using the bicinchoninic acid (BCA) protein assay following the manufacturer's directions (Therma Fisher Scientific, Rockford, IL). Protein extracts were stored at −80°C until use.

### 2.5. Determination of IL-1*β* in Heart Homogenates

Commercially available ELISA kit (R&D Systems, Abingdon, UK) was used to measure concentrations of IL-1*β* in tissue homogenates, according to the manufacturer's instructions.

### 2.6. Western Blot Analysis

Equal amounts of total protein extracts were separated by SDS-PAGE and electrotransferred to nitrocellulose membrane (GE-Healthcare Europe, Milan, Italy). Membranes were probed with rabbit anti-NLRP3 (Abcam, Cambridge, UK), rabbit anti-caspase-1 (Santa Cruz Biotechnology, Dallas, TX, USA), mouse anti-Ser^473^Akt (Cell Signaling Technology, Danver, MA, USA), rabbit anti-total Akt (Cell Signaling Technology, Danver, MA, USA), rabbit anti-Ser^9^ GSK-3*β* (Abcam, Cambridge, UK), anti-total GSK-3*β* (Cell Signaling Technology, Danver, MA, USA), anti-Ser^660^ PKC and total PKC (Santa Cruz Biotechnology, Dallas, TX, USA), SOD2 (Novus Biologicals, Centennial, CO, USA), and NRF2 (Thermo Fisher Scientific, Whaltam, MA, USA) followed by incubation with appropriate HRP-conjugated secondary antibodies (BioRad). Proteins were detected with Clarity Western ECL substrate (BioRad, California, USA) and quantified by densitometry using analytic software (Quantity-One, BIO-RAD Image Lab Software.6.0.1.). Results were normalized with respect to densitometric value of mouse anti-tubulin (Abcam, Cambridge, UK), and autoradiograms showing statistically significant differences in terms of gel-loading homogeneity were excluded from the following biomarkers analyses.

### 2.7. Platelet Release of S1P and Adenosine

Fasting venous blood sample from four male healthy volunteers (mean age: 38 ± 2 years) was withdrawn without stasis and anticoagulated with citrate-dextrose solution (ACD, with the final ACD/blood ratio 1: 6 vol/vol). The human platelet study was authorized by “Comitato Etico Interaziendale San Luigi Gonzaga,” authorization *n.* 155/2017, and informed consent was obtained in accordance with the 1964 Declaration of Helsinki and its later amendments. The platelet-rich plasma, obtained by centrifugation at 100 × g for 20 min, underwent further centrifugation at 2000 × g for 10 min, and pellet was washed 2 times at 37°C in HEPES-Na buffer (mmol\L): 10 HEPES Na, 140 NaCl, 2.1 MgSO_4_, 10 D-glucose, and pH 7.4. Platelets were counted by automatic blood cells counter (Mythic 18, Orphèe, Switzerland) and resuspended to a final concentration of 2 × 10^11^ cells/L in phosphate-buffered saline containing 1% BSA. The contamination of white blood cells was less than 1/10^4^ platelets. Platelet samples were subjected to stirring (1200 rpm speed at 37°C) in both the absence and presence of Ticagrelor (5000 ng/mL, 30 min) or thrombin receptor-activating peptide (TRAP-6) (Mascia Brunelli, Monza, Milan, Italy) (10 *μ*mol/L, 8 min), then centrifuged at 4000 rpm for 10 min. Supernatants were stored at -20°C until sphingosine, S1P, and adenosine measurements.

### 2.8. Sample Preparation for UHPLC-Tandem Mass Analysis

100 *μ*L of platelet samples and heart homogenates were added with 2 mL of 0.1% trifluoroacetic acid in chloroform/methanol 1/1 and with internal standards (adenosine and S1P d7) at 300 *μ*g/L as final concentration. After vortex in g for 30 seconds, 0.5 mL of chloroform and 0.5 mL of water were added. After centrifugation, organic phase was recovered and extracted twice with 1 mL of chloroform. The solution was dried overnight under vacuum (Centrivap, Labconco, Kansas City, MO, USA) and reconstituted with 100 *μ*L of eluents A/B 7/3.

### 2.9. UHPLC-Tandem Mass Analytical Method

The analyses of sphingolipids and adenosine were performed using a Nexera (Shimadzu, Milan, Italy) UHPLC coupled through an ESI source to a Qtrap5500 triple quadrupole analyzer (Sciex, Milan, Italy).

The chromatographic separation was achieved with a Kinetex column (1.7 *μ*m, 100 × 2.1 mm, 100 Å, Phenomenex, Bologna, Italy) with 0.1% formic acid in water/acetonitrile 8/2 (eluent A) and 0.1% formic acid in isopropanol/acetonitrile 8/2 (eluent B). The separation gradient was from 5 to 100% of B in 7 minutes, followed by column reconditioning. Flow rate was set at 400 *μ*L min^−1^, and injection volume was 3 *μ*L. The LC column effluent was delivered to the ESI ion source, using air as both 1 and 2 gasses (40 and 50 arbitrary units, respectively), and the ion voltage was 5.0 kV. Curtain gas (nitrogen) was 30 arbitrary units.

The multiple reaction monitoring (MRM) transitions and parameters were C18-Sph (*m*/*z*) 300@282 CE 13 V; C18-S1P (*m*/*z*) 380@264 CE 21 V; and adenosine (*m*/*z*) 268@136 CE 21 V. For the internal standard, the MRM transition was C18-S1P d7 (*m*/*z*) 387@271 CE 19 V. The lower limit of detection (LLOQ) was 0.50 *μ*g/L for all analytes.

### 2.10. Materials

Unless otherwise stated, all compounds were purchased from the Sigma-Aldrich Company Ltd. (St. Louis, Missouri, USA).

### 2.11. Statistical Analysis

All values are expressed as means ± SEM and were analyzed by ANOVA test followed by Bonferroni's posttest and Student's *t*-test. A *P* value < 0.05 was considered statistically significant.

## 3. Results

### 3.1. Infarct Size Was Reduced by Ticagrelor In Vivo but Not Ex Vivo

**Figure 2:**
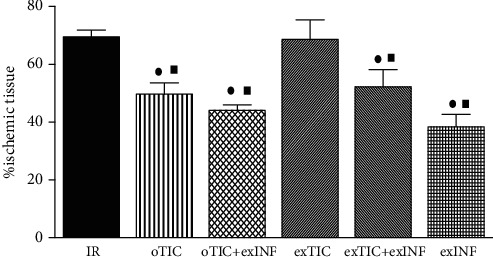
Infarct size. Data analyzed by one-way ANOVA followed by a Bonferroni post hoc test and expressed as mean ± SEM. *n* = 6–8 per group. Statistical significance: ^●^*P* < 0.05 vs. IR and ^■^*P* < 0.05vs. exTIC.

Rat hearts exposed to a 30 min global ischemia and 60 min reperfusion developed infarction which was 69.5 ± 2.3% of ischemic area at risk (AAR). Infarct size was significantly reduced by rat pretreatment for 3 days with Ticagrelor (oTIC, infarct size 49.6 ± 3.9% of AAR, *P* < 0.01 vs. IR group). No protective effects were recorded when Ticagrelor was added *ex vivo* to the perfusate of excised hearts from untreated animals (exTIC, infarct size 68.6 ± 3.0% of AAR), thus suggesting that Ticagrelor protection was only triggered in the intact organism (Figure 2). Coronary flow and perfusion pressure measured during stabilization in the IR group (10 ± 1 mL/min/g and 80 ± 2 mmHg, respectively) were not statistically different from those recorded in the treated groups, thus suggesting similar oxygen demands among groups.

### 3.2. Ticagrelor and INF Do Not Exert Additive Effects on Infarct Size Limitation

When INF was added *ex vivo* to the perfusate before ischemia (exINF), we observed a significant reduction in infarct size (exINF, infarct size 38.3 ± 3.0% of AAR, *P* < 0.05 vs. IR group), comparable to the one achieved by oTIC alone (Figure 2).

Interestingly, exINF did not exert additive effects on protection against infarct size evoked by oral Ticagrelor pretreatment, as the reduction in infarct size evoked by the combination was almost identical to that obtained herein with either Ticagrelor or exINF alone (oTIC+exINF, infarct size 44.1 ± 1.9% of AAR, *P* < 0.05 vs. IR group). Similarly, no priming effects on exINF protection were recorded when Ticagrelor was coadministered *ex vivo* in the perfusate only (exTIC+exINF, infarct size 52.2 ± 2.7% of AAR).

### 3.3. Ticagrelor Pretreatment Prevented NLRP3 Inflammasome Activation and Downstream Signaling

Expression level and activation of the downstream signaling of NLRP3 inflammasome were assessed by Western blotting analysis in protein extracts obtained from the apical portion of hearts pretreated or not with either Ticagrelor or INF and exposed to IR.

**Figure 3:**
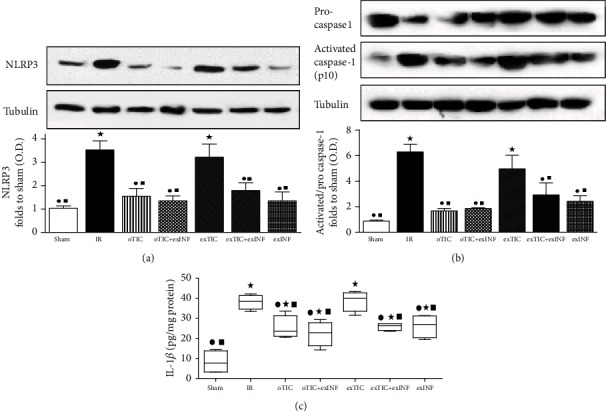
Western blotting analysis on (a) NLRP3, (b) pro- and activated caspase, and (c) quantification of IL1*β* by ELISA kit assay. Data analyzed by one-way ANOVA followed by a Bonferroni post hoc test and expressed as mean ± SEM. *n* = 6–8 per group. Statistical significance: ^●^*P* < 0.05 vs. IR; ^★^*P* < 0.05 vs. sham, ^■^*P* < 0.05 vs. exTIC. Representative blots are shown of at least three different experiments.

As expected, the INF pretreatment effectively reduced the IR-induced NLRP3 upregulation and activation, resulting in significant reduction of the cleaved active p10 subunit of caspase-1 (Figures 3(a) and 3(b)). As a consequence of reduced caspase-1 activation, the levels of IL-1*β*, that reached the highest concentrations after 60 min of reperfusion, showed a mild but still significant decrease in the exINF group (Figure 3(c)). Notably, both Western blotting analysis and ELISA assay demonstrated that similar inhibition of NLRP3 expression and activation could be reached when rats were pretreated with Ticagrelor (oTIC), but not when Ticagrelor was added in the perfusate only (exTIC). Besides, no further NLRP3 inflammasome inhibition was recorded when INF was added in the perfusate of heart from rats previously exposed to Ticagrelor pretreatment (oTIC+exINF).

### 3.4. Risk Pathway Protective Activity Was Enhanced by Either Oral Ticagrelor Pretreatment Or INF Heart Exposure

**Figure 4:**
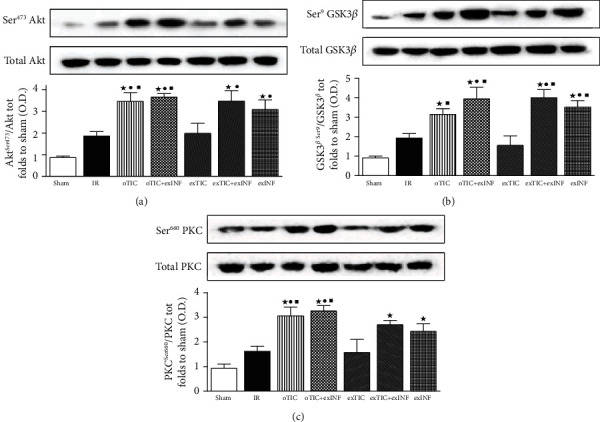
Western blotting analysis on (a) Akt, (b) GSK3*β*, and (c) PKC. Data analyzed by one-way ANOVA followed by a Bonferroni post hoc test and expressed as mean ± SEM. *n* = 6–8 per group. Statistical significance: ^●^*P* < 0.05 vs. IR; ^★^*P* < 0.05 vs. sham, ^■^*P* < 0.05 vs. exTIC. Representative blots are shown of at least three different experiments.

Since RISK pathway is activated by both pre- and postconditioning treatments [34], we quantified expression and activity (in terms of phosphorylation) of its key members. After 60 min of reperfusion, slight but not significant increase in phosphorylation rate of Akt, GSK-3*β*, and PKC (Figures 4(a)–4(c), respectively) was recorded in untreated hearts exposed to IR protocol, when compared to the sham group. The phosphorylation rates of Akt, GSK-3*β*, and PKC induced by the 60 min reperfusion were all increased massively in oTIC and exINF (both *P* < 0.05 vs. sham; *P* = NS among groups). No additive effects were recorded when the two treatments were combined. Interestingly, *ex vivo* Ticagrelor exposure 20 min prior to ischemia only did not significantly modify the activation of the RISK pathway evoked by IR and/or INF.

### 3.5. Ticagrelor and INF Improved IR-Induced Antioxidant Response

**Figure 5:**
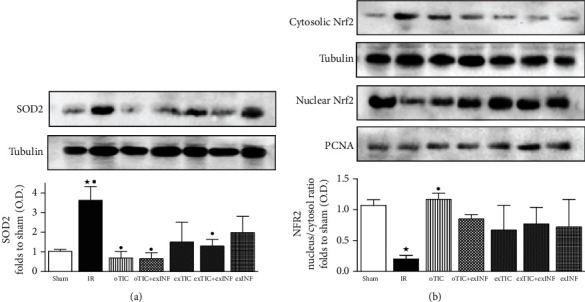
Western blotting analysis on (a) SOD2 and (b) nuclear and cytosolic Nrf2. Data analyzed by one-way ANOVA followed by a Bonferroni post hoc test and expressed as mean ± SEM. *n* = 6–8 per group. Statistical significance: ^●^*P* < 0.05 vs. IR; ^★^*P* < 0.05 vs. sham, ^■^*P* < 0.05 vs. exTIC. Representative blots are shown of at least three different experiments.

SOD2 is an important endogenous antioxidant and provides protection against myocardial IR. Consistently with other studies [24], we found that IR led to increased expression of SOD2 (*P* < 0.05 vs. sham). All treatments blunted IR-induced SOD upregulation. However, in oTIC, oTIC+exINF, and exTIC+exINF groups, the levels of SOD2 were significantly lower than that of IR group (*P* < 0.05 vs. IR, Figure 5(a)). A reduction in SOD2 levels was also recorded in the heart of mice treated with INF only when compared to IR group, without reaching statistical significance (Figure 5(a)).

The Western blot analysis on the expression levels of the antioxidant transcription factor Nrf2 showed that its nuclear translocation was reduced by IR when compared to sham (Figure 5(b)). All treatments blunted IR-induced reduction of Nrf2 nuclear translocation. However, only in oTIC the levels of Nrf2 nuclear translocation were significantly higher than in IR and similar to sham (Figure 5(b)).

### 3.6. Oral Ticagrelor Pretreatment Resulted in S1P and Adenosine Overaccumulation in the Heart

**Figure 6:**
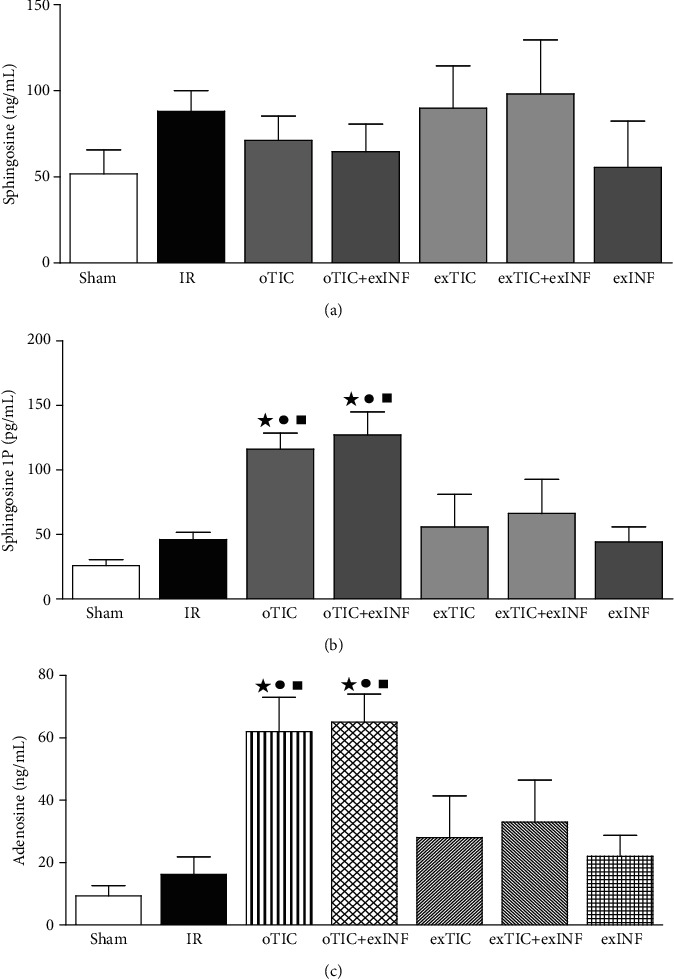
Myocardial levels of sphingosine, sphingosine 1P (S-1P), and adenosine. Data analyzed by one-way ANOVA followed by a Bonferroni post hoc test and expressed as mean ± SEM. *n* = 6–8 per group. Statistical significance: ^●^*P* < 0.05 vs. IR; ^★^*P* < 0.05 vs. sham, ^■^*P* < 0.05 vs. exTIC.

As shown in Figure 6, a marked increase in the myocardial concentration of either S1P or adenosine was recorded in the postischemic heart from rats exposed to the oral Ticagrelor pretreatment (oTIC) when compared to IR only, whereas *ex vivo* administration of Ticagrelor or exINF did not significantly affect their levels. Combining Ticagrelor and INF (oTIC+exINF) did not further increase S1P or adenosine accumulation in the heart in comparison to oTIC alone.

### 3.7. Ticagrelor Enhanced S1P and Adenosine Release from Platelets

Sphingosine, S1P, and adenosine were evaluated in the supernatant of human platelet samples subjected to stirring (1200 rpm speed at 37°C) with or without Ticagrelor (5000 ng/mL, 30 min) (Table 1). Ticagrelor-treated samples show high level of S1P in the supernatant and low level of sphingosine if compared to control (*P* < 0.05). Adenosine concentration increased significantly compared to control in the supernatant of Ticagrelor-treated platelets. The positive control was obtained by incubating human platelet with TRAP-6 (10 *μ*mol/L, 8 min). The values of the release of S1P and adenosine in the supernatant in TRAP-6 group were significantly higher than control and Ticagrelor-treated platelets (data not shown).

## 4. Discussion

The present study further extends previous findings on Ticagrelor cardioprotective effects, confirming that the protection was dependent upon its administration *in viv*o, as adding the P2Y12 antagonist *ex vivo* to the perfusate in excised hearts does not counteract the IR injury. Here, we confirm our previous data [24] that the specific and direct inhibition of NLRP3 by exINF results in a significant reduction in infarct size. Most notably, adding INF just before ischemia does not further improve cardioprotection induced by Ticagrelor pretreatment (3 days), with no significant effect of the combination over each drug alone. We then aimed to assess whether Ticagrelor primes the isolated hearts exposed to the inflammasome inhibitor. Administration of Ticagrelor to the perfusate *ex vivo* does not enhance the heart response to exINF, showing no interactions between the two treatments.

Indeed, Ticagrelor has been shown to modulate the expression on blood cells of toll-like receptors, key receptors involved in NLRP3 regulation [35]. Here, we show that the reduction in infarct size achieved by oral Ticagrelor is, at least in part, attributable to a cardioprotective effect mediated by the inhibition of the NLRP3 inflammasome pathway, as similar inhibitory effects on the activation of pivotal markers of the inflammasome cascade were recorded when either pharmacological tools (oral Ticagrelor or exINF) were used. With these two treatments, there is also an upregulation of the RISK pathway and a limitation of IR-induced oxidative stress.

To the best of our knowledge, so far, only another study has suggested that cardioprotection of Ticagrelor tested in models of acute myocardial injury can be partially attributable to inhibition of mRNA levels of NLRP3 and IL-1beta in the heart of diabetic rats [13]. Here, we extended these observations to nondiabetic conditions and we documented that Ticagrelor, when administered to rats *in vivo*, evokes significant decrease of protein levels of NLRP3, resulting in lower activation of caspase-1, thus counteracting the IR-induced accumulation of active IL-1beta proteins in the heart.

A pharmacological approach with cardioprotective inhibitors has suggested that cardioprotection induced by P2Y12 antagonists is due to a conditioning phenomenon rather than to their antiplatelet effect [6]. In the presence of sphingosine kinase inhibitor, Cangrelor's antiplatelet effect seems intact; nevertheless, *in vivo* studies did not definitively rule out a contribution from the antiplatelet effect in limiting IR injury. Our study, in which the P2Y12 antagonist was administered *in vivo* and myocardial infarct subsequently induced *ex vivo*, in the absence of platelets, definitively confirms that the protective effect is mainly due to a preconditioning effect *in vivo* that lasts throughout the IR procedure *ex vivo*. The lack of cardioprotection by Ticagrelor when administered in the *ex vivo* model further support the idea of a blood cell-mediated preconditioning effect [5, 8, 31]. Given the apparent dependence upon the presence of blood in mediating Ticagrelor effects [5, 17], it would seem logical to propose that the cardioprotective effect of Ticagrelor is dependent on platelets and likely the P2Y12 receptor. This is supported by previous observations showing that chemically distinct P2Y12 antagonists have similar cardioprotective properties [6, 16, 17]. We thus investigated the ability of Ticagrelor to affect platelet ability to release S1P, an essential, bioactive lysophospholipid mediator that regulates various physiological functions such as lymphocyte trafficking, inflammation, and behavioural characteristics of the vascular system [36]. Platelets are among the major source of S1P in the circulation [17, 37], and platelet-derived S1P has been demonstrated to exert a critical role in the repair of pivotal microvascular structures during injury [38, 39]. Ticagrelor is unique in being an inhibitor of equilibrative nucleotide transporter 1 (ENT1) [6, 13, 14, 16]. Here, we confirm these effects in isolated human platelets, as revealed by an increase in S1P and adenosine release after exposure to Ticagrelor. Ticagrelor was previously demonstrated to raise tissue levels of adenosine, which is a known endogenous cardioprotective substance in pathophysiological conditions of the heart, including myocardial ischemia and heart failure [40, 41]. This effect has been suggested to involve inhibition of ENT1 in heart tissue [13–15]. However, so far, no experimental evidence of direct effects of Ticagrelor on ENT1 has been reported. Besides, our findings on the lack of cardioprotection by Ticagrelor on the isolated heart cloud this hypothesis of cardioprotection through interference with cardiac ENT 1 [8]. Thereby, its effects on increasing adenosine levels in heart tissue could derive from an effect of Ticagrelor on a subgroup of blood cells. Both erythrocytes and platelets are known to release different substances in blood stream, including adenosine and S1P [17, 42, 43]. Here, we demonstrated for the first time that Ticagrelor increased the levels of adenosine released from platelets, thus suggesting that this effect might contribute, together with S1P, to the cardioprotection recorded when Ticagrelor was administered *in vivo* only.

The contribution of blood-derived S1P and adenosine in mediating the myocardial protective effects of Ticagrelor was confirmed by showing that both S1P and adenosine reached the highest concentrations in the heart of rats orally exposed to Ticagrelor. While the increase in myocardial adenosine levels has been already documented in the IR heart of rats orally pretreated with Ticagrelor [13], so far, no direct myocardial detection of S1P or comparison of adenosine/S1P myocardial levels between *in vivo* vs. *ex vivo* treatments has been reported in the literature. Thus, our study adds a further interesting piece of evidence on the ability of Ticagrelor to cause blood cells to release substances, which may contribute to cardioprotection. In fact, either S1P or adenosine has been already demonstrated to evoke protective effects throughout activation of the protective survival RISK pathway in the heart [44, 45]. As Ticagrelor protection seems to depend at least in part on the same signaling cascade modulate S1P and adenosine, it seemed likely that these endogenous components would be involved in Ticagrelor's protective mechanism. Interestingly, both the treatment (Ticagrelor and exINF) in aerobic conditions do not affect myocardial perfusion, thus suggesting an unchanged oxygen demand in comparison to untreated hearts. Moreover, the levels of components of either NLRP3 or RISK pathways in sham animals were lower than those detected in all IR groups (protected and nonprotected), thus suggesting that in aerobic conditions, the pharmacological treatments do not influence the myocardial metabolism, but may trigger mechanisms that will make the hearts more resistant to IR challenge boosting RISK activation in postischemic phase. Indeed, the RISK pathway is an intrinsic prosurvival signaling cascade evoked by IR itself which confers protection against the reperfusion insult by avoiding the opening of the mitochondrial permeability transition pore at the onset of reperfusion [34, 46]. Potentiation of RISK activation in the early minutes of reperfusion contributes to cardioprotection induced by preconditioning protocols. Actually, in protected hearts, the phosphorylation of RISK enzymes peaks at 10-15 minutes of reperfusion and progressively wanes thereafter [34, 47–49]. A two-threefold higher phosphorylation level after 60 min of reperfusion is a strong indication of kinase involvement in protection, especially if we consider that the reduction in infarct size in protected hearts resulted in a ~20% increase in vital tissue when compared to control IR hearts. As previously documented [6], pharmacological inhibition of the RISK pathway blunted the protective effects of P2Y12 antagonists against IR, thus further supporting the hypothesis that their mechanisms of cardioprotection utilize specific signal transduction of myocardial protection rather than inhibition of intravascular coagulation. Our study extends these findings confirming cross-talk mechanisms linking NLRP3 inflammasome to RISK pathway in cardioprotection, which have been so far faintly suggested [50–52], but not convincingly demonstrated. Besides, it shows that Ticagrelor uses similar mechanisms of protection evoked by a NLRP3 inflammasome inhibitor, leading to activation of the RISK pathway. The lack of additive effects of the drug combination may be explained considering that additional protection cannot be induced by strategies that share common prosurvival signaling pathways such as the P2Y12 antagonist and the NLRP3 inflammasome inhibitor. However, previous studies demonstrated that treatment with a caspase-1 inhibitor prior to ischemia or reperfusion adds its protection to the one elicited by the P2Y12 antagonist, Cangrelor [4, 7]. Differently from INF that directly targets NLRP3 complex formation, direct caspase-1 inhibitors may influence not only inflammatory response but also glycolytic, mitochondrial, and pyroptotic cell death [4]; thus, the additional beneficial effects recorded by these authors could be due to interference with any of these pathways beyond the inhibition of the NLRP3 inflammasome-caspase axis. Another substantial difference between our study and that of Audia et al. [4] is that these authors had higher coronary flow when the caspase-1 inhibitor was used, while we perfused the hearts at constant flow, to avoid flow effects on IR injury.

The role of oxidative stress in contributing to IR injury is not clear [53, 54]. Overall, it seems that IR-dependent oxidative stress is reduced by all protective treatments. However, only Ticagrelor pretreatment displays a consistent effect in limiting the oxidative component as suggested by the significant increase in nuclear levels of Nrf2 and by the downregulation of SOD2. Nevertheless, also for this mechanism, there are not apparent differences between Ticagrelor and INF. Moreover, the antioxidant effect of Ticagrelor given orally seems stronger than that observed when it is given *ex vivo*.

## 5. Limitation of the Study

Our results confirm Ticagrelor conditioning effects, which are not additives to the cardioprotection achieved by directly inhibiting NLRP3. It is likely that this lack of additive effect is due to the activation of RISK pathway by both treatments. Other studies demonstrated additive effect when P2Y12 and a downstream NLRP3 factor, namely, caspase-1, were inhibited [4, 7], thus confirming that the cross-talk between NLRP3 and RISK cardioprotective pathways is quite complex [36]. Besides, here, we did not test the impact of the proposed pharmacological treatments on the tested signaling cascades at basal condition. Therefore, further studies are needed to fully elucidate the cross-talk among mechanisms linking NLRP3 complex, redox state, and RISK pathway. Finally, we must consider that all experimental paradigms have disadvantages and advantages. For instance, we used gavage to administer Ticagrelor instead of a spontaneous intake which is more physiological, as gavage guarantees a more constant dosage, which is recommendable in cardioprotection studies [55]. Determining which blood-derived factors mediate Ticagrelor-induced cardioprotection was beyond the scope of this study. Nevertheless, the fact that Ticagrelor increases platelet release of both adenosine and S1P suggests these factors as important players that deserve further investigations.

## 6. Conclusions

In conclusion, we confirm that Ticagrelor requires the presence of blood to act as conditioning agent. Importantly, we demonstrate that the cardioprotective effects of Ticagrelor are not due to a direct action on the myocardial tissue nor to its antiaggregating effect, whereas the NLRP3 inhibitor, INF, is able to act directly on the heart. Nevertheless, these two drugs given before ischemia activate a similar protective pathway, involving RISK pathway and redox modulation, without additive cardioprotective effects.

## Figures and Tables

**Table 1:** Level of sphingosine, sphingosine 1P (S-1P), and adenosine released by platelets exposed to Ticagrelor.

	Sphingosine (ng/mL)	S-1P (ng/mL)	Adenosine (ng/mL)
Control	356.9 ± 44.3	94.3 ± 29.0	18.8 ± 5.8
Ticagrelor	68.4 ± 2.4^∗^	300.7 ± 38.5^∗^	48.8 ± 5.4^∗^

Level of sphingosine, S-1P, and adenosine, measured with UHPLC-tandem mass analysis, in the supernatant of human platelet subjected to stirring (1200 rpm speed at 37°C) incubated in absence and presence of Ticagrelor (5000 ng/mL, 30 min). Data analyzed by Student's *t* test and expressed as mean ± SEM; *n* = 6 − 8 per group in duplicate. Statistical significance: ^∗^*P* < 0.05 vs. control.

## Data Availability

The data used to support the findings of this study are available from the corresponding author (MC and PP) upon request.

## Abbreviations

RISK: Reperfusion Injury Salvage Kinase
IR: Ischemia/reperfusion
AMI: Acute myocardial infarction
NLRP3: Nucleotide-binding oligomerization domain- (NOD-) like receptor pyrin domain containing 3
IS: Infarct size
AAR: Area at risk
INF: NLRP3 inflammasome inhibitor.
